# Laser Sintered Magnesium-Calcium Silicate/Poly-ε-Caprolactone Scaffold for Bone Tissue Engineering

**DOI:** 10.3390/ma10010065

**Published:** 2017-01-13

**Authors:** Kuo-Yang Tsai, Hung-Yang Lin, Yi-Wen Chen, Cheng-Yao Lin, Tuan-Ti Hsu, Chia-Tze Kao

**Affiliations:** 1 Department of Oral and Maxillofacial Surgery, Changhua Christian Hospital, Changhua 500, Taiwan; 72837@cch.org.tw; 2 Department of Oral and Maxillofacial Surgery, China Medical University Hospital, Taichung 40447, Taiwan; forevermarch1@hotmail.com; 3 Graduate Institute of Biomedical Sciences, China Medical University, Taichung 40447, Taiwan; evinchen@gmail.com; 4 3D Printing Medical Research Center, China Medical University Hospital, China Medical University, Taichung 40447, Taiwan; roger.line0204@gmail.com (C.-Y.L.); nakohsu@gmail.com (T.-T.H.); 5 School of Dentistry, Chung Shan Medical University, Taichung 40201, Taiwan; 6 Department of Stomatology, Chung Shan Medical University Hospital, Taichung 40201, Taiwan

**Keywords:** laser sintering, calcium silicate, scaffold, osteogenesis, human marrow stem cells

## Abstract

In this study, we manufacture and analyze bioactive magnesium–calcium silicate/poly-ε-caprolactone (Mg–CS/PCL) 3D scaffolds for bone tissue engineering. Mg–CS powder was incorporated into PCL, and we fabricated the 3D scaffolds using laser sintering technology. These scaffolds had high porosity and interconnected-design macropores and structures. As compared to pure PCL scaffolds without an Mg–CS powder, the hydrophilic properties and degradation rate are also improved. For scaffolds with more than 20% Mg–CS content, the specimens become completely covered by a dense bone-like apatite layer after soaking in simulated body fluid for 1 day. In vitro analyses were directed using human mesenchymal stem cells (hMSCs) on all scaffolds that were shown to be biocompatible and supported cell adhesion and proliferation. Increased focal adhesion kinase and promoted cell adhesion behavior were observed after an increase in Mg–CS content. In addition, the results indicate that the Mg–CS quantity in the composite is higher than 10%, and the quantity of cells and osteogenesis-related protein of hMSCs is stimulated by the Si ions released from the Mg–CS/PCL scaffolds when compared to PCL scaffolds. Our results proved that 3D Mg–CS/PCL scaffolds with such a specific ionic release and good degradability possessed the ability to promote osteogenetic differentiation of hMSCs, indicating that they might be promising biomaterials with potential for next-generation bone tissue engineering scaffolds.

## 1. Introduction

Bone tissue is also known as spongy or cancellous structure and is a porous, reticulate osseous tissue [1]. New kinds of bone substitute are being developed by inducing osteogenic and angiogenic differentiation to meet the requirements for bone regeneration, and with specific bioactive molecules this has stimulated great interest in recent years. Therefore, bone implant materials have shown great potential for hard tissue regeneration via cell behavior simulation and calcified tissue formation [2,3,4]. Calcium phosphate-based biomaterials, such as *β*-tricalcium phosphate and hydroxyapatite, due to their generally good biocompatibility, osteoconductivity and similarity to the inorganic component of natural bone have been broadly used in biomedical applications [5,6]. Moreover, bioactive materials have large surface areas that can be active and directly bonded to the natural bone tissues. Calcium silicate (CS)-based materials have received great attention in recent years due to their excellent bioactivity when compared to calcium phosphate-based materials [7]. Recently, various researchers demonstrated CS-based materials could have assisted in hard tissue formation and regeneration because of the Si ion release and fast apatite precipitation ability of CS-based material [7,8,9]. In our lab, a fast-setting CS cement was successfully produced with a mixture of CaO, SiO_2_, and Al_2_O_3_, which showed a significant setting time reduction [10,11]. The CS materials showed good osteoconduction and inhibited inflammation in primary human dental pulp cells (hDPCs). Moreover, previous reports show that CS-based materials are able to promote osteogenic and odontogenic differentiation from various types of cells [12,13,14]. However, the low degradation rate of CS-based materials may result in an inhibition in osteoconductivity, which may inhibit the clinical applications [15]. Therefore, the Mg doped into CS cement was analyzed in a previous study, and the degradation of materials may be controlled by the Mg contained [13]. After immersion in SBF for 3 months, weight losses of 10.2% and 41.5% were measured for pure CS and 20% Mg-contained CS, respectively, which indicates a significant difference.

The introduction of polymer into ceramics to fabricate composite materials was always to enhance the Young’s modulus and strength of the materials while maintaining its biocompatibility [16]. However, the degradable biopolymers could be employed as an infiltrated matrix which initially supported the structural integrity but subsequently degraded to supply porous construction necessary for cell migration and tissue regeneration [17]. Polycaprolactone (PCL) was the Food and Drug Administration (FDA)-approved material for several medical and drug delivery devices and now is extensively used for tissue regeneration owing to its cost-effectiveness, durability, excellent biocompatibility and biodegradability [18]. It has also attracted more attention, as it can be easily processed into scaffolds with various shapes because of its relative low melting point (55–60 °C) and good blend compatibility with other inorganic additives [19,20]. Various studies confirmed PCL as a usable material for fabrication of 3D macroporous scaffolds, specifically for bone tissue regeneration [21]. Moreover, 3D-printed PCL scaffolds have been tested both in vitro and in vivo, demonstrating their use as bone substitutes in critical-size defects [22,23]. However, PCL has a long degradation due to its semi-crystallinity and hydrophobicity [24]. 

Currently, the most common 3D printing technology to produce composite scaffolds is fused deposition modeling (FDM), which is a rapid prototyping technique that can manufacture desired 3D structures using a computer-aided design model. FDM has been used to fabricate various types of tissue engineering scaffolds [25,26]. However, the composites fabricated via FDM always mixed several polymers or binders with the organic solvents. Therefore, this process may damage the cells, so the use of tissue engineering is not a good choice. Selective laser sintering (SLS) is a versatile powder-based 3D printing system via particle-bonding, which provides the most optimal solution for reconstructive-based bone tissue engineering [24]. Many biocompatible polymers have been investigated for SLS, such as PCL, polyvinyl alcohol and poly-lactide [24,27,28]. In addition, the laser power had a great effect on the sintering degree of ceramic phase, which led to better mechanical properties between particles which would result in higher strength of 3D-scaffolds [27]. Most importantly, the SLS method does not need the use of organic solvents, which produced organic–inorganic composite scaffold geometries, and does not form the filament (as in fused deposition modeling) [29,30]. It may be easier to fabricate multiple materials for the fabrication of new tissue engineering 3D-scaffolds.

Based on 3D printing technology, it has been developed to manufacture more ideal structure scaffolds with better control of pore morphology, shape and porosity [31]. Thus, 3D printing can be used to build different a versatile, solid, free-form microenvironment supplying unprecedented flexibility in both material and geometry, a potential way to create customized scaffolds for tissue ingrowth [32]. The aim of this study was to investigate whether the Mg–CS/PCL hybrid 3D scaffold supplies a suitable microenvironment for the osteogenetic differentiation of human mesenchymal stem cells (hMSCs) and to further consider the effect of the Mg–CS/PCL composite on the biological performance of hybrid scaffolds.

## 2. Materials and Methods

### 2.1. Preparation of Mg–CS Powder

The method used here for the preparation of Mg–CS powder has been described elsewhere [15]. In brief, reagent grade CaO (Sigma-Aldrich, St. Louis, MO, USA), SiO_2_ (High Pure Chemicals, Saitama, Japan), MgO (Sigma-Aldrich) and Al_2_O_3_ (Sigma-Aldrich) powders were used as matrix materials (composition: 60% CaO, 20% SiO_2_, 15% MgO, and 5% Al_2_O_3_). The oxide mixtures were sintered at 1400 °C for 2 h in a high-temperature furnace. In this study, 0%, 10%, 20%, and 30% of Mg–CS was mixed with PCL powder for scaffold fabrication. The Mg–CS and PCL were ball-milled in ethyl alcohol using a centrifugal ball mill (S 100, Retsch, Hann, Germany) for 12 h and dried at 50 °C for 12 h. The specimen codes ‘CS0’, ‘CS10’, ‘CS20’ and ‘CS30’ stood for the specimens containing 0 wt % Mg–CS/100 wt % PCL, 10 wt % Mg–CS/90 wt % PCL, 20 wt % Mg–CS/80 wt % PCL and 30 wt % Mg–CS/70 wt % PCL, respectively.

### 2.2. Scaffold Fabrication

The Mg–CS/PCL 3D scaffolds were fabricated using a system built in the lab, which contained CO_2_ laser fiber (2 W). DesignX software was used to plan the dimensions and structure distribution of Mg–CS/PCL scaffolds. Each specimen had 16 layers of 500 µm layer distance, 500 µm line distance and a combined height of 8.0 mm and a base diameter of 10 mm. Air blowing was used to clean the powder without adhering in the pores of scaffolds.

### 2.3. Characterization

The water contact angle for each specimen was determined at room temperature. Briefly, specimens were placed on the top of a stainless steel base, a drop of MilliQ water (10 μL) was placed on the surface of the specimens, and the image was taken by a camera after 20 s had elapsed. The resulting images were analyzed using ImageJ (National Institutes of Health) to consider the water contact angle. The concentration of the measured elements was given in atomic percent. The phase composition of the cements was analyzed using X-ray diffractometry (XRD; Bruker D8 SSS, Bruker, Karlsruhe, Germany) and run at 30 kV and 30 mA with a scanning speed of 1°/min. The specimens were coated with gold and their morphologies were investigated under a scanning electron microscope (SEM; JSM-6700F, JEOL) operated in the lower secondary electron image (LEI) mode at 3 kV accelerating voltage. In addition, the Mg–CS content of the scaffolds was determined by thermogravimetric analysis (TGA, STA 449C, Netzsch, Bavaria, Germany). The samples were analyzed in aluminum pans under a nitrogen purge and heated from 0 to 700 °C with a heating rate of 10 °C/min.

### 2.4. Immersion Test

The bioactivities of the Mg–CS/PCL scaffolds were considered by examining the formation of bone-like apatite on the specimens in simulated body fluid (SBF) solution. The scaffolds with a thickness of 10 mm and a diameter of 8 mm were immersed in SBF at 37 °C in a humidified atmosphere containing 5% CO_2_ for various time-points with a surface-area-to-volume ratio of 0.1 cm^2^/mL without refreshing SBF. After various immersion time-points, the specimens were gently rinsed with ddH_2_O to remove SBF and dried in vacuum at 50 °C. The surfaces of the immersed samples were observed by SEM. The Ca, Si, Mg, and P ion concentrations released from composites on SBF were analyzed using an inductively coupled plasma-atomic emission spectrometer (ICP-AES; Perkin-Elmer OPT 1MA 3000DV, Shelton, CT, USA).

### 2.5. Cell Adhesion and Proliferation

Before performing the cell experiments, all scaffolds were sterilized by immersion in 75% EtOH and exposure to UV light for 20 min. The human marrow mesenchymal stem cells (hMSCs) were obtained from Sciencell Research Laboratories (Sciencell, Carlsbad, CA, USA) and grown in commercial mesenchymal stem cell medium (Sciencell) at passage 3–6. After different culturing times, cell viability was determined by the PrestoBlue^®^ (Thermo Fisher Scientific Inc., Waltham, MA, USA) assay. At the end of the culture period, the medium was discarded and the wells were washed twice with cold PBS. Each well was filled with a 1:9 ratio of PrestoBlue^®^ in fresh DMEM and incubated at 37 °C for 1.5 h. The solution in each well was then transferred to a new 96-well plate and Tecan Infinite 200^®^ PRO microplate reader (Tecan, Männedorf, Switzerland) was used at 570 nm with a reference wavelength of 600 nm. Cells cultured on tissue culture plates without materials were used as a control (Ctl). The results were obtained in triplicate from three separate experiments in terms of optical density (OD).

### 2.6. Western Blot

After 3 h of culture, cells on scaffolds were lysed with NP40 buffer (ThermoFisher). The total protein concentrations were determined using BCA protein assay kit. The cell lysates (40 μg protein) were separated using sodium dodecyl sulfate-polyacrylamide (SDS)-polyacrylamide gel electrophoresis and then transferred to PVDF membranes (Millipore, Billerica, MA, USA). After blocking with 2% BSA in TBST for 1 h, the membranes were immunoblotted with the primary anti-pFAK, anti-FAK, and *β*-actin (GeneTex, San Antonio, TX, USA) for 2 h. The bands were then visualized after incubation for 1 h with horseradish peroxidase (HRP)-conjugated secondary antibodies by chemiluminescence using an ECL detection kit (Invitrogen, Carlsbad, CA, USA). The protein expression level was normalized to the *β*-actin for each group.

### 2.7. Osteogenesis Assay

The osteogenetic differentiation medium was StemPro Osteogenesis Differentiation Kit from Invitrogen. The level of ALP activity was determined after the cells had been seeded for 3 and 7 days. The process was as follows: the cells were lysed from discs using 0.2% NP-40 and centrifuged for 10 min at 2000 rpm after washing with PBS. ALP activity was determined using p-nitrophenyl phosphate (pNPP, Sigma) as the substrate. Each sample was mixed with pNPP in 1 M diethanolamine buffer for 15 min, after which the reaction was stopped by the addition of 5 N NaOH and quantified by absorbance at 405 nm. All experiments were done in triplicate. The osteogenic differentiation medium was Advance MEM (Thermo Fisher Scientific Inc.) supplemented with 10^−8^ M dexamethasone (Sigma-Aldrich), 0.05 g/L l-Ascorbic acid (Sigma-Aldrich) and 2.16 g/L glycerol 2-phosphate disodium salt hydrate (Sigma-Aldrich). The OC protein released from the hMSCs was cultured on different substrates for 7 and 14 days after cell seeding. Following the manufacturer’s instructions, an osteocalcin enzyme-linked immunosorbent assay kit (Invitrogen) was used to determine the OC protein content. The OC protein concentration was measured by correlation with a standard curve. The analyzed blank disks were treated as controls. All experiments were done in triplicate.

### 2.8. Alizarin Red S

The accumulated calcium deposition was analyzed using Alizarin Red S staining, following a method developed for a previous study [22]. In brief, the specimens were fixed with 4% paraformadedyde (Sigma-Aldrich) for 15 min and then incubated in 0.5% Alizarin Red S (Sigma-Aldrich) at pH of 4.0 for 15 min at room temperature. Then, the photographs were observed using an optical microscope (BH2-UMA; Olympus, Tokyo, Japan) equipped with a digital camera (Nikon, Tokyo, Japan) at 200 magnifications. After this, the scaffolds were washed with PBS and quantified using a solution of 20% methanol and 10% acetic acid in water. After 15 min, the liquid was transferred to a 96-well plate, and the quantity of Alizarin Red was determined using a spectrophotometer at 450 nm.

### 2.9. Statistical Analysis

A one-way variance statistical analysis was used to evaluate the significance of the differences between the groups in each experiment. Scheffe’s multiple comparison test was used to determine the significance of the deviations in the data for each specimen. In all cases, the results were considered statistically significant with a *p* value <0.05.

## 3. Results and Discussion

### 3.1. Characterizations of Mg–CS/PCL Scaffold

**Figure 1:**
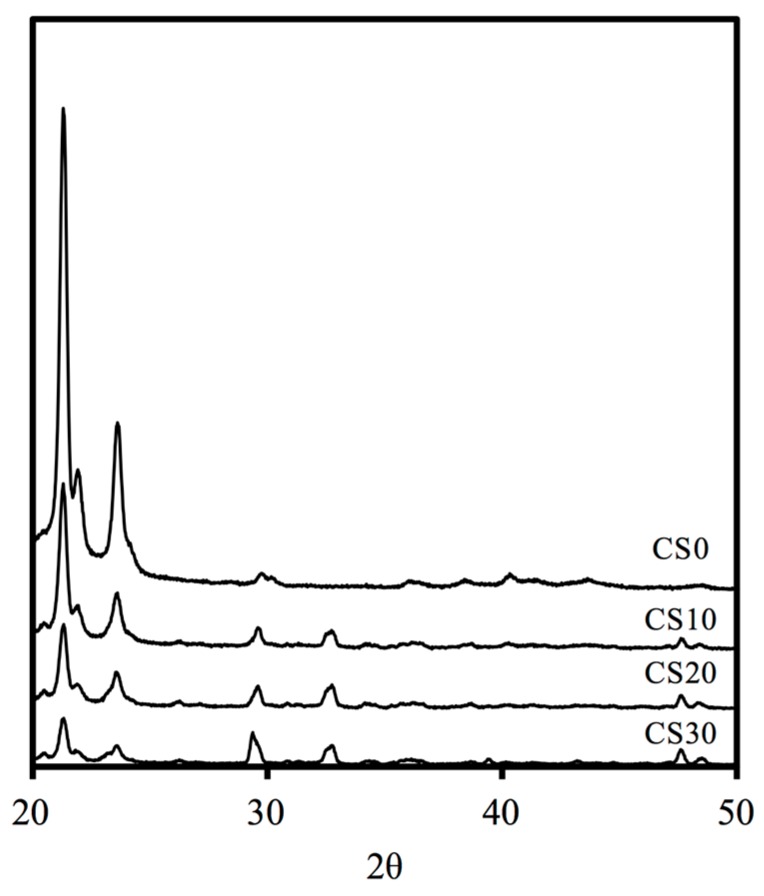
XRD patterns of the various Mg–CS content of 3D scaffolds.

The X-ray diffractometry analysis shows that the diffraction peaks at 21.44°, 22.06°, 23.76°, and 36.32° are typical for PCL (Figure 1). The presence of these narrow peaks makes sense when we consider the semicrystalline nature of this polymer [33]. The diffraction peaks at 2θ = 29.4°, which corresponds to the CSH gel and incompletely reacted inorganic component phases of the Mg–CS at 2θ between 32° and 34° [34]. This confirms that Mg–CS has been incorporated into the PCL. The reduction in the intensity of the peaks is due to microstructural changes in PCL and changes in its crystallinity.

**Figure 2:**
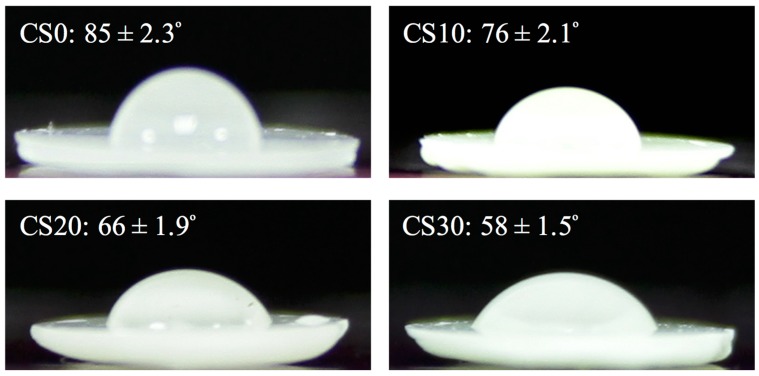
Water contact angle of different scaffolds.

Wettability is the key factor to consider when designing bone substitutes because it plays a key role in determining biological behavior [22]. Figure 2 shows the contact angle of the Mg–CS/PCL surfaces. The contact angle decreases as the Mg–CS contained is raised, with contact angles of 85° ± 2.3°, 76° ± 2.1°, 66° ± 1.9° and 58° ± 1.5° for the CS0, CS10, CS20 and CS30 groups, respectively. The decrease in contact angle after coating led to the increased hydrophilicity of the material surface. The cell behavior can be promoted if grown on scaffolds with a water contact angle in this range; the result show that CS0 scaffold is hydrophobic, while a scaffold mixed with Mg–CS precipitate is extremely hydrophilic [35].

**Figure 3:**
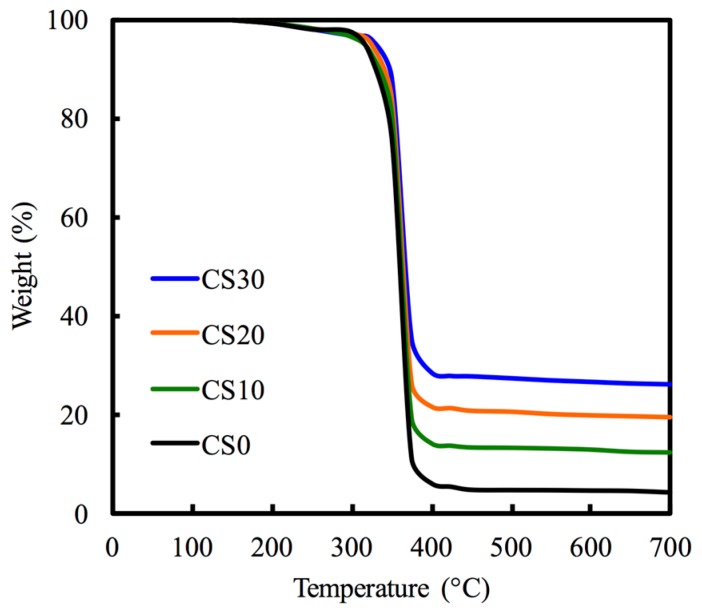
TGA curves of different Mg–CS/PCL scaffolds.

Figure 3 indicates the thermogravimetric analysis results used to examine the content of Mg–CS within the scaffolds. As showed in the thermograms, the maximum rate of weight loss occurred when the temperature was approximately 410 °C for all scaffolds, which actually corresponds to the burned PCL phase. The remaining weights of CS0, CS10, CS20, and CS30 were 4.51%, 12.31%, 19.51%, and 26.33%, respectively.

### 3.2. Bioactivity

**Figure 4:**
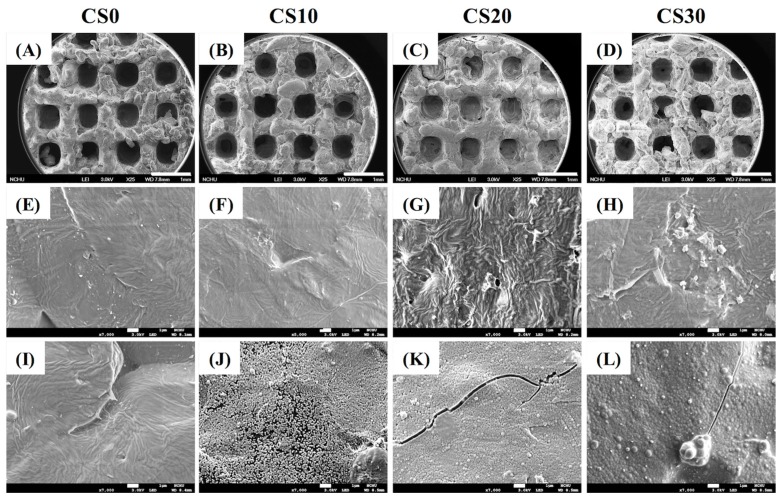
Surface SEM images of the scaffolds (**A**–**D**) before immersion in SBF—low magnification; (**E**–**H**) before immersion in SBF—high magnification; and (**I**–**L**) after immersion in SBF.

SEM was utilized to analyse the morphology and microstructure of the scaffolds (Figure 4). The macropores with an interconnected structure design were clearly visible in all scaffolds. In all scaffolds (Figure 4A–D), the pore size was about 450 µm, which has the potential to influence new bone regeneration and vascularization [36]. The results of apatite precipitation on scaffold before (Figure 4E–H) and after soaking in SBF for 1 day (Figure 4I–L) were considered by morphology. After immersion in SBF for 1 day, there was no apatite precipitation on the CS0 specimens. Evidently, increasing Mg–CS contained induced more apatite precipitation on the surface of Mg–CS/PCL scaffolds, which is in good agreement with the SEM results (Figure 4L). The formation of the bone-like apatite in SBF has proven to be useful in predicting the bone-bonding ability of material in vitro. Presumably, the apatite-forming ability of the materials is dependent on the Ca–Si ratio of the specimens. The Si–OH functional groups on the surface of calcium silicate-based materials have been shown to act as the nucleation center for apatite precipitation [9]. As reported in a previous study [37,38,39], the release of Ca ions, possibly originating from the less-ordered hydration products, could significantly promote apatite growth by promoting local Ca supersaturation, thereby increasing the ionic product of the apatite in the surrounding environment and promoting the nucleation rate of the apatite. After immersion for 1 day, the surface structure of CS20 and CS30 was uniformly covered with spherical aggregated minerals with an average size of approximately 1 μm. However, there was little apatite precipitate on the CS10 surface (Figure 4J). 

**Figure 5:**
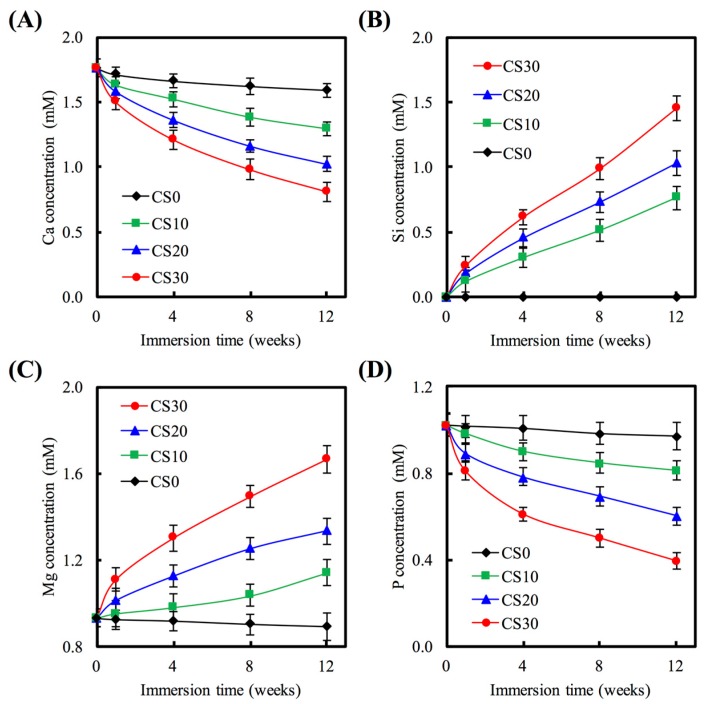
(**A**) Ca; (**B**) Si; (**C**) Mg; and (**D**) P concentration of SBF after immersion for different time.

Figure 5 shows the Ca, Si, Mg, and P ion concentrations after being soaked for different time periods in SBF. For CS0, the Ca ion concentration of SBF was approximately 1.71 mM after immersion for 1 week (Figure 5A), which was not significantly different compared to 0 week (*p* > 0.05). For CS30, the Ca ion concentration of the solution was significant lower (*p* < 0.05) than CS0 for all time points. The amount of Si that was released from each of the scaffolds is presented in Figure 5. B. Si is continuously released during the soaking from 1 to 12 weeks, indicating that the CS30 are slowly released Si ion over the entire period of the immersion process. After CS0, CS10, CS20, and CS30 soaked in SBF for 12 weeks, the Si ion concentration of the solution was 0, 0.76, 1.30, and 1.45 mM, respectively. For Mg ions, it is obvious that the concentration released from the scaffold was increased when Mg–CS increased (Figure 5C). It is already well-established that Mg ions play an important role in the health of the human body, can enhance DNA and protein synthesis and indirectly regulates mineral tissue metabolism [40]. In recent years, Mg–CS have been reported in hard tissue regeneration, which can been control the degradation rate and mechanical properties with excellent bioactivity [13]. Chen et al. proved that the Mg ion released from Mg–CS not only exhibited no cytotoxicity against the human cell in vitro, but also enhanced cementogenesis and angiogenesis of human periodontal ligament cells [15]. In contrast, P ions significantly declined at the groups containing Mg–CS for all time points (Figure 5D). Moreover, the reduction in the concentration of Ca and P ions that might result in the precipitate of the apatite layer. This results indicated that CS30 with an optimum bioactivity was expected to form a chemical bond between natural bone and implanted biomaterials through the thick apatite layer.

**Figure 6:**
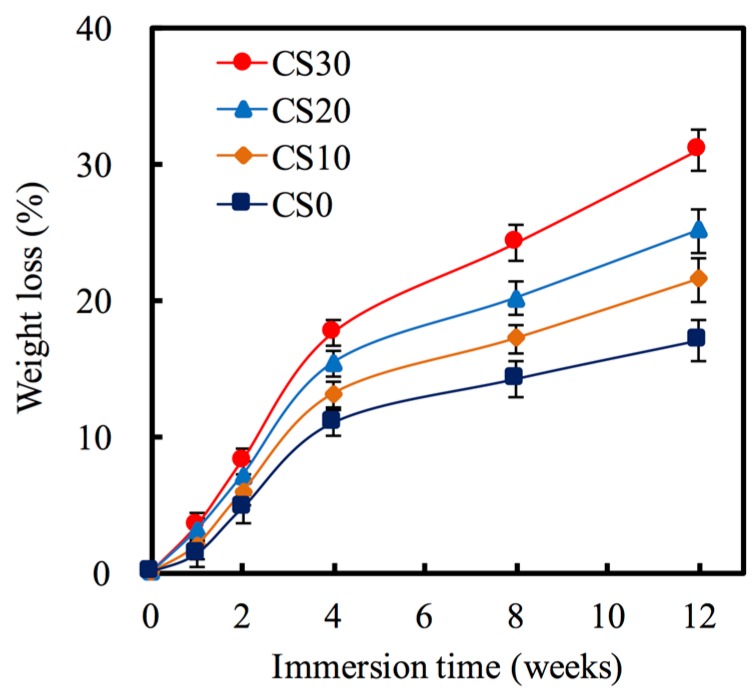
Weight loss of various scaffolds after immersion in SBF for predetermined time durations.

The degradation behavior of the Mg–CS/PCL in SBF solution was evaluated for various periods of time ranging from 1 week to 12 weeks. In Figure 6, the CS0 shows much less degradation than the other scaffolds, exhibiting a weight loss of 11.02% after immersion in the SBF for 4 weeks. At the final time-point of the soaking, weight losses of approximately 17.06%, 21.48%, 25.09%, and 31.01% were observed for the CS0, CS10, CS20, and CS30 scaffolds, respectively, indicating significant differences (*p* < 0.05). It is well known that biomaterials with various degradation rates are required for different clinical uses [41,42]. Thus, we found the Mg amount in CS-based material will influence the degradation rate of materials. We think that the higher degradation rate of the scaffolds with higher Mg–CS content was due to the large surface area and pore volume compared to the CS0 and CS10 [13].

### 3.3. Cell Adhesion

**Figure 7:**
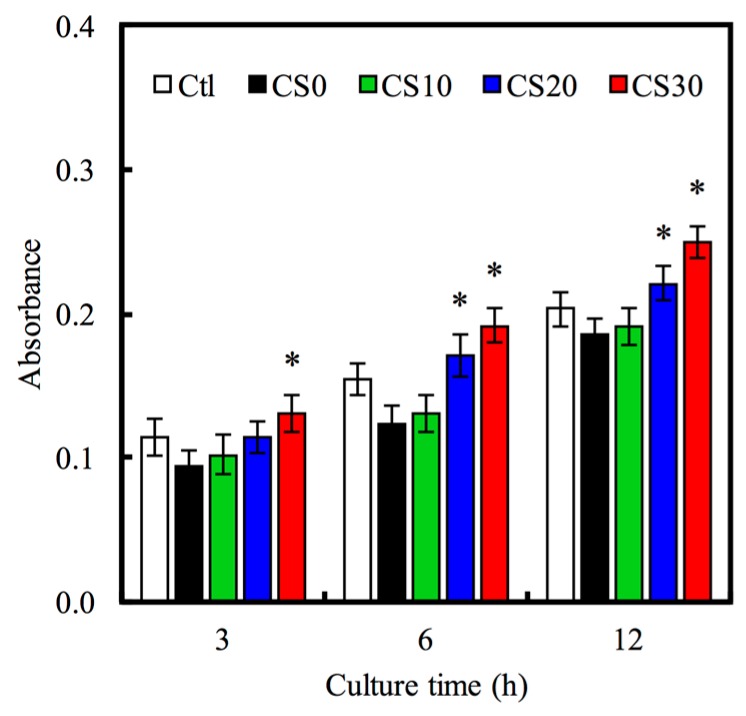
The adhesion of hMSCs cultured with various specimens for different time-points. “*” indicates a significant difference (*p* < 0.05) compared to CS0.

**Figure 8:**
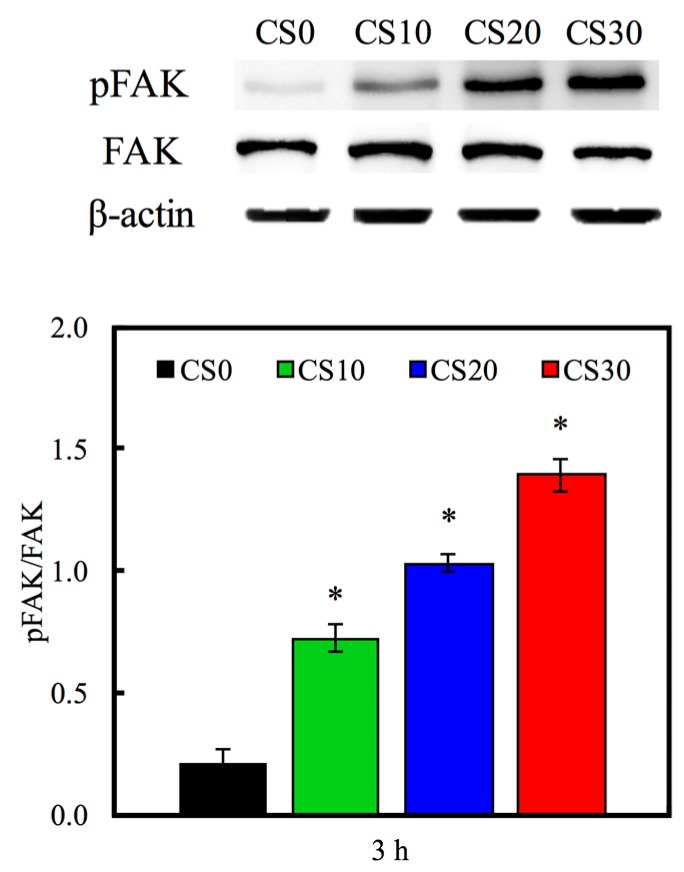
Western blotting of pFAK protein expression of hMSCs cultured on various specimens for 3 h. “*” indicates a significant difference (*p* < 0.05) compared to CS0.

The hMSCs adhering to the Mg–CS/PCL hybrid 3D-scaffolds were evaluated by the PrestoBlue^®^ assay in Figure 7. The results show the absorbance of the PrestoBlue^®^ treatment with hMSCs cultured on the CS30 for 3, 6 and 12 h was significantly higher (*p* < 0.05) than CS0 specimens compared and the normal tissue culture plate (Ctl) for all culture periods time points. This result indicates that the Mg–CS contained scaffolds might facilitate hMSCs adhesion. In addition, cells adhering to substrates is essential to maintain biological function and mechanical integrity. These interactions are regulated by trans-membrane cell–cell and cell–matrix interaction molecules [43]. Focal adhesion kinase (FAK) is one mediator of downstream signaling that reportedly regulated cell adhesion, migration, and differentiation in various cells [44,45]. To consider FAK involvement in Mg–CS/PCL regulation of cell adhesion, we cultured hMSCs on the various materials. The Western blot result (Figure 8) indicated that pFAK was significantly increased on Mg–CS contained scaffolds compared with pure PCL (CS0), that more than 6.8-times on CS30 compared to CS0. FAK can be activated by integrin-mediated with ECM-related proteins, and this results in the phosphorylation of FAK. Increased phosphorylation of FAK has been shown in various primary cells [46]. In a previous study, the dental pulp cells and hMSCs cultured on CS materials clearly showed that cell adhesion preferred Si-rich groups and adsorbed more ECM-related proteins [46]. The amount of ECM proteins influences cell adhesion via integrin-mediated adhesion behavior on the different biomaterial surfaces [47]. Thus, we suggested the mechanical stimulus generated by the interaction between the Mg–CS/PCL surface and cells was transformed into the signaling transduction via FAK that played a role in the following cell adhesion and osteogenesis differentiation.

### 3.4. Cell Proliferation

**Figure 9:**
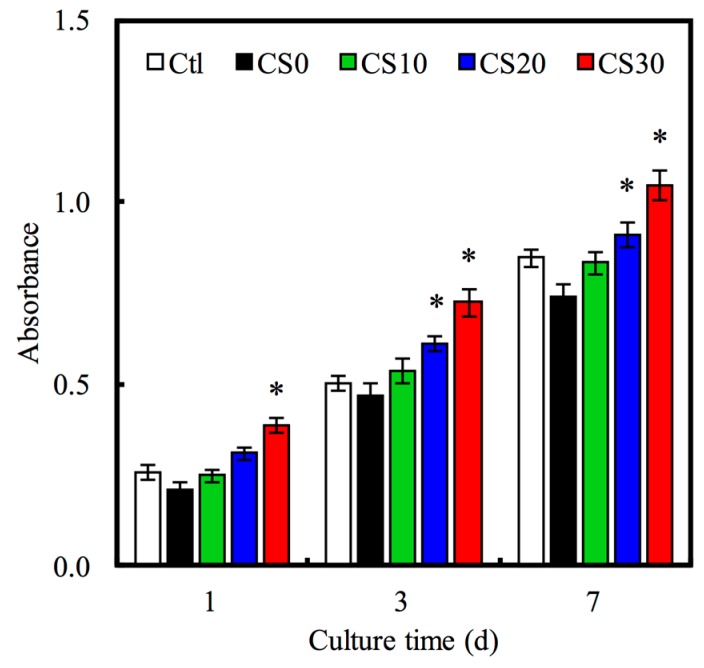
The proliferation of hMSCs cultured with various specimens for different time-points. “*” indicates a significant difference (*p* < 0.05) compared to CS0.

Furthermore, the cell proliferation assays for 1, 3 and 7 days were performed with PrestoBlue^®^ cell viability reagents and are shown in Figure 9. The addition of Mg–CS enhanced the proliferation rate of hMSCs compared with pure PCL (CS0). Meanwhile, the cell proliferation of hMSCs in CS30 specimen was obviously higher than that in CS0 and CS10 specimens throughout the whole culture time (*p* < 0.05). These results proved that the proliferation of hMSCs on the scaffolds was obviously promoted by the increase in Mg–CS content, suggesting that incorporation of Mg–CS into PCL could significantly promote hMSCs proliferation. The hydrophilic surface of CS30 was favorable for cell behavior, such as attachment and spreading. Furthermore, the rich Mg^2+^ ions of the CS30 specimens played the important role of surface ligands for hMSC-favored ECM adsorption, which was beneficial for biocompatibility. We partially attribute this enhancement to the release of Si ions from Mg–CS produced by the degradation of Si-containing materials (Bioglass, MTA), and direct cell cultures on Si-contained materials have also achieved excellent results on hMSCs [48,49].

### 3.5. Osteogenetic Differentiation

**Figure 10:**
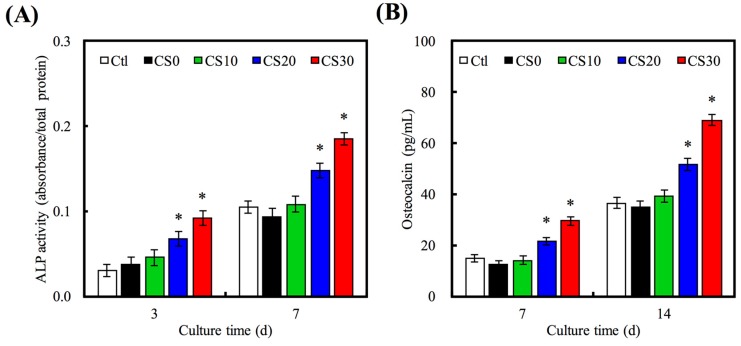
(**A**) ALP activity; and (**B**) OC amount of hMSCs cultured on various scaffolds for different time points. “*” indicates a significant difference (*p* < 0.05) compared to CS0.

**Figure 11:**
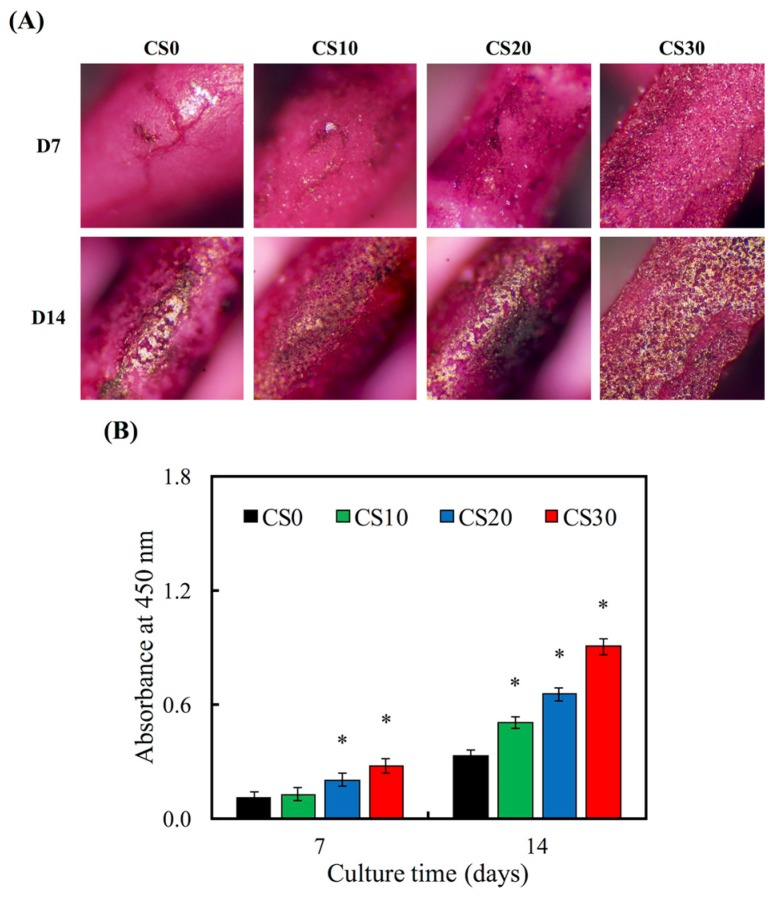
(**A**) Alizarin red S staining and (**B**) quantification of calcium mineral deposits by hMSCs cultured on Mg–CS/PCL for 1 and 2 weeks. The “*” indicates a significant difference (*p* < 0.05) compared to CS0.

ALP is an enzyme found mostly in the bone cells and is also an early osteoblastic differentiation marker. The ALP activity of hMSCs for 3 and 7 days is shown in Figure 10A. The ALP activity increased with time and Mg–CS concentration. Furthermore, the ALP activity of hMSCs cultured on CS20 and CS30 scaffolds was significantly higher (*p* < 0.05) than Ctl at 3 and 7 days. OC is the most abundant non-collagenous protein in bone and is also important in bone metabolism. It is used as a clinical marker for bone turnover. The protein expression level of OC on hMSCs was measured by ELISA (Figure 10B). On day 7 and 14, the significant difference (*p* < 0.05) between higher Mg–CS concentration (CS20 and CS30) and Ctl was found. In addition, the Alizarin Red S staining was used to evaluate calcium deposits in cell culture (Figure 11). When increasing the concentration of Mg–CS, the amounts of calcium mineral deposits increased and the color of Alizarin Red S staining ranged from light to deep pink. These results were similar to pure CS bone cements [10]. They indicated that the Mg–CS/PCL scaffolds can enhance the expression level of the osteogenic differentiation markers and might stimulate calcium deposition and mineralized nodule formation, and suggest these Mg–CS/PCL scaffolds have great potential for promoting the osteogenic differentiation of hMSCs. Shie et al. proved that calcium silicate could stimulate the adhesion, proliferation and differentiation of hMSCs and the ERK1/2 pathway might play an important role in the modulation of these effects [43]. Dong et al. demonstrated that Si-based nanomaterials have the potential to promote osteogenetic differentiation of hMSCs, which could enhance the ALP activity and the expression of osteogenetic differentiation-related genes including ALP, BSP and OC [50].

## 4. Conclusions

In conclusion, the 3D scaffolds with controlled macropores on the surface were prepared by combining the 3D printing technique with the Mg–CS/PCL hybrid strategy. In this study, we have succeeded in fabricating uniform scaffolds containing PCL and Mg–CS using laser sintering. Moreover, as compared to CS0 scaffolds without an Mg–CS powder, the hydrophilic properties and degradation rate were also improved. Interestingly, the Mg and Si ions released from Mg–CS-containing scaffolds significantly enhanced the proliferation and increased the osteogenic-related protein of hMSCs compared with pure PCL. We think the simple fabrication process for Mg–CS/PCL scaffolds combined with their excellent physical and biological ability suggests that scaffolds containing Mg–CS have great potential for bone tissue engineering applications, and particularly for the regeneration of large bone defects.
